# Book Review: W. H. FINCH, J. E. Bolin and K. Kelley: Multilevel Modeling Using R: 2nd Edition, CRC Press, 2019, 252 pp, ISBN: 9781138480674

**DOI:** 10.1007/s11336-021-09812-y

**Published:** 2021-10-23

**Authors:** Nivedita Bhaktha

**Affiliations:** GESIS - Leibniz Institute for the Social Sciences, B6 4-5, 68159 Mannheim, Germany

Multilevel modeling, also known as hierarchical or mixed effects modeling, is a statistical technique used for fitting nested or clustered data. Multilevel modeling aids in examining associations between variables measured at different levels of the data structure (Raudenbush & Bryk, 2002; Hox et al., 2018). In these models, residual components are introduced at each level of the structure. Multilevel modeling is a popular method of analysis and is widely used in social sciences (students nested within classrooms), natural sciences (burrowing habit across locations), physical sciences (distribution of light over the sky), and medicine (tracking drug doses over time). In recent years, there has been a lot of research on modeling clustered data for methods such as structural equation modeling (SEM), mediation analysis, mixture modeling, propensity score analysis to name a few. Due to the ubiquity of this method, all major statistical softwares offer implementations of multilevel modeling. Especially, R has become widely popular among researchers and teachers for fitting multilevel models. With the development of multilevel modeling in research and software, it is crucial that researchers acquaint themselves with the method and learn to implement it in R.

There are several books on multilevel modeling that offer technical and non-technical treatment of the subject. Some books also offer guidance on fitting these models using different software. There are a couple of excellent books that include implementation of multilevel modeling in R—linear mixed effects models using R (Galecki & Burzykowski, 2013) and mixed effects models and extensions in Ecology with R (Zuur et al., 2009). Yet, this book is a welcome addition on the topic. The most important aspect of this book is the communication of the fundamentals of multilevel modeling and some of its advanced issues in a non-technical and easy to understand format. The comprehensive description of R functions and syntax makes it easier for a novice R user to fit multilevel models and understand the resulting output. The website associated with the first edition of the book (http://www.mlminr.com/) contains data sets used in the examples that can be downloaded for practice by the readers. The second edition of the book includes examples with newer packages, functions, model fit measures, and additional advanced topics, but the above-mentioned website has not been updated to accommodate the changes. However, the book contains all the necessary R codes and syntax to replicate the examples.

*Primary audience of the book:* This book is mainly geared toward readers in need for an introduction to multilevel models as the book does not delve into advanced topics or technical details of the methodology. However, seasoned researchers and course instructors trying to include R implementations will also find this book to be useful. The book covers a broad range of themes and examples, making it easier for readers from varied background to follow the content.

*Structure of the book:* The book is well written and structured. It covers a wide range of topics related to multilevel modeling and communicates the basics lucidly. Readers do not need to have a background in advanced or generalized linear models, as the authors have explained the basic principles wherever necessary. Each chapter in the book follows a set format which starts with an abstract of the chapter and is then followed by a brief introduction of the topic in a non-technical voice. Next, important concepts are highlighted through illustrative examples along with a description of R functions, options, and syntax. Finally, each chapter ends with a summary. Initial chapters explain the foundation of multilevel linear models and its implementations in R (Chapters 1–3). After providing the basics, the next part of the book (Chapters 4–8) deals with extension of multilevel models to higher levels and non-normal responses. The topics include higher level models, longitudinal analysis, graphing multilevel data structure, and multilevel generalized linear models. The last part of the book (Chapters 9–10) briefly describes advanced topics in multilevel analysis such as Bayesian modeling, outlier detection, robust estimation, lasso, and power analysis.

*Summary of the book:* Chapter 1 starts with a brief introduction to linear modeling and succinctly covers topics such as categorical predictors and model assumptions testing. Chapters 2 & 3 lay the foundation of multilevel modeling. Chapter 2 briefly describes the impact of nested data and the pitfalls of ignoring such a structure on the results and inference. This chapter defines models with the random intercept and slope and briefly touches on the issues of centering, estimation, and assumptions. Chapter 3 demonstrates how to fit a two level linear model using *lmer()* function from the *lme4* package. Chapter 4 extends the implementation of multilevel modeling to three and higher level structures. It clearly outlines the basic framework and syntax for higher level models. Chapter 5 is very brief and slightly diverges from the topics up to that point and explains the use of multilevel framework for longitudinal (person-period) data analysis. Chapter 6 introduces graphs for multilevel models using *lattice* and *effects* package. Chapter 7 provides an introduction to generalized single-level linear models. It quickly recaps logistic, multinomial, ordinal, and count regression models and describes the use of* glm()* function from *MASS* package for fitting them. Chapter 8 is an extension of Chapter 7 to a multilevel setting using *glmer()* function for fitting the models. All examples in this chapter are limited to a two level setting. Chapter 9 is a standalone section on Bayesian multilevel modeling. While this chapter assumes that readers have at least a rudimentary understanding of Bayesian analysis, the examples are easy to follow and the authors have explained some of the aspects of Bayesian analysis using *MCMCglmm()* function. The last chapter deals with advance issues of multilevel modeling such as robust estimation, lasso, generalized additive models to name a few. This chapter barely scratches the surface of the topics covered.

*Fitting models in R* One of the strengths of this book is that it makes fitting multilevel models in R approachable to novice R users. The book provides explanation of each R function and syntax used in the examples. This helps the readers understand what is being done by the function and why it is needed. For certain topics, the authors discuss the implication of default options and the additional options that are available for the function. For example, the authors mention that *lme4* does not report p-values, but they provide a quick fix by suggesting the usage of *confint()* function to obtain bootstrapped confidence interval. Such information is advantageous to beginners interested in fitting multilevel models in R. However, for certain other topics, such as graphing multilevel data and Bayesian analysis, popular and most widely used packages and functions are not even mentioned. Researchers already familiar with multilevel analysis and looking to shift to R will also find this book to be a good resource.

*Limitations and recommendations* The title of the book makes one expect a thorough treatment of the method in R—both in terms of breadth and depth of the content. The authors have valued breadth over depth in this presentation. This is not a big disadvantage given that the authors have mentioned in the preface (of the first edition) that the objective of this book is to serve as a guidebook that will encourage the readers to launch their own investigation into multilevel modeling. However, the authors do not provide any recommended reading list or set of resources at the end of each chapter for the readers to delve deeper into certain topics. Additional textbooks, articles, and R tutorials are required if the readers want to rigorously implement multilevel models in R. Addition of recommended reading lists on—(1) technicalities of method and (2) application of method, would be helpful to the readers. Inclusion of examples on presenting the results of multilevel models for articles and reports would also be beneficial. Some of the presentation on advanced topics are extremely terse and barely manage to scratch the surface. The last chapter can be confusing and overwhelming for beginners. Instead, certain other topics commonly encountered in social sciences such as interaction effects and cross-classified models might have been beneficial for beginners. Exercises or practice problems for the readers to solve in R would also strengthen the book as a self-learning resource.

In conclusion, the book is a useful first line resource for a researcher beginning to fit multilevel models in R. Overall, the book is well written and can serve as a guidebook for multilevel modeling. To a large extent, the book fulfils its goal of serving as a self-learning tool for beginners. Despite its minor shortcomings, the book has made the content and implementations of multilevel models in R, approachable for beginners.
